# Professor Dickson on the Intermarriage of the Races

**Published:** 1845-07

**Authors:** 


### PROFESSOR DICKSON ON THE INTERMARRIAGE OF THE RACES.

We have received from the accomplished author, Professor Dickson, of Charleston, a pamphlet in which this subject is ably discussed. It is chiefly in reply to a letter from the eminent physiologist, Dr. Carpenter, of England, maintaing that the barriers between the African and Caucasian races are not impassable, and that the former, in time, may be raised to an equality with the latter. Dr. Dickson insists that the intercourse, by which it is proposed to effect this improvement in the African race, would end in introducing a mongrel breed, superior indeed to the negro, but vastly inferior to the whites; and in this way, although one family of the species would be raised on the scale of humanity, the entire race would be degraded. He makes a strong argument, and adduces facts which must puzzle his adversaries.

Dr. C. believes, with Dr. Nott, that the mulatto is incapable of "keeping up his number," but must decay and disappear. We thought Dr. Forry's refutation of this doctrine was triumphant; but

Dr. D. is persuaded that time will establish its correctness. He has no doubt of the comparative infertility of mulattoes, or of the inferior average duration of life among them. Statistics must decide the question; and so far as they have been collected Forry showed that they favored the contrary opinion.

Dr. Dickson refers to Mexico and Egypt as affording illustrations of the effects of crossing the breeds. In the former it is said to be hard to find "a white under twenty years of age of unmixed descent," the difficulty being to decide upon the proportion in each of the old Spaniard, the Morisco, the Indian and the Negro. The Doctor thinks "we would scarcely select the Mexican as a choice specimen of humanity." He is right. Of the ancient land of civilization and the arts, he says:

"In regard to Egypt, the influx of negroes is estimated at 3,000 a year—anciently by Arrian, recently by Madden—Morton calculates the number introduced within 3,500 years at more than 10,000,000. Clot Bey states the present negro population at 20,000; adding, that negresses form the greater number of women in every harem.

"Thus we account at once for the degeneracy of the modern compared with the ancient Egyptian—and for the progressive depopulation of that fertile and once densely peopled and highly civilized country." Y.

